# Coefficient Estimates for New Subclasses of Bi-Univalent Functions Involving Generalized Bivariate Fibonacci-like Polynomials

**DOI:** 10.12688/f1000research.173513.2

**Published:** 2026-04-28

**Authors:** Mustafa Husseinu, Mohammed H. Saloomi

**Affiliations:** 1 University of Kerbala, Karbala, Karbala Governorate, Iraq; 2 University of Kerbala, Karbala, Karbala Governorate, Iraq

**Keywords:** Regular function, univalent function, subordination, bi-univalent function, Fibonacci-like polynomials functions, Fekete- Szegö

## Abstract

The study of bi-univalent functions plays an important role in geometric function theory, particularly in determining coefficient bounds for analytic functions. Despite significant progress, there remains a need to develop broader subclasses that allow for more flexible analytical frameworks. Motivated by this, the present work introduces new subclasses of bi-univalent functions defined via generalized bivariate Fibonacci-like polynomials.

The proposed classes are constructed using subordination principles and suitable functional relations associated with these polynomials. Based on this approach, estimates for the initial coefficients are derived for functions belonging to the defined subclasses. In addition, Fekete–Szegö inequalities are established, extending several existing results in the literature.

The findings demonstrate that the use of generalized Fibonacci-type structures provides an effective tool for obtaining sharper coefficient bounds. These results contribute to the ongoing development of the theory and may serve as a basis for further investigations in related subclasses of analytic and bi-univalent functions.

## 1. Introduction

Many polynomials including Fibonacci and Bell Many polynomials are widely used in many researches in the context of this topic in addition to Lucas polynomials and Horadam polynomials. Other special polynomials also exist. Most of these polynomials are employed in some branches comprising geometry, physics, and theoretical analysis such as. ^1– 4^ In addition, they are utilized in the theory of geometric functions and number theory, for example, Refs. 5– 7, and this is what interests us in these polynomials. In the context of the topic, the principle of subordination and quasi -subordination has been used in complex analysis, especially in the geometric functions theory. After forming new subclasses of regular functions, many researchers have used polynomials and estimated the initial coefficients and the Fekete-Szegö problem. Based on the relationship between polynomials and the classes of regular functions, the two new classes are defined.

After defining these two subclasses and calculating the upper bounds of the coefficients, the researchers concluded many important results and observations, after using the basic rules of polynomials.

Through the recurrence relation:

$${℣}_{n}={℣}_{n-1}+{℣}_{n-2},{℣}_{0}=0,{℣}_{1}=1,\left(n\geq 2\right).$$

The Fibonacci polynomials are a polynomial sequence that may be regarded as a generalization of the Fibonacci numbers. In Ref. 8, a new generalization of the Fibonacci bolynomials called generalized bivariate Fibonacci-like is introduced.

Concerning *n* ≥ 2, the recurrence relation:

$${℣}_{n}\left(x,y\right)=px{℣}_{n-1}\left(x,y\right)+\mathrm{qy}{℣}_{n-2}\left(x,y\right)$$

is possible to define the generalized bivariate Fibonacci-like polynomials where p , q represent positive integers while “ x,y∈ℝ , ${℣}_{0}\left(x,y\right)=a,{℣}_{1}\left(x,y\right)=b,px,\mathrm{qy}\neq 0\;\text{and}\;{\left(px\right)}^{2}+4\mathrm{qy}\neq 0\;$ ”.

For the Fibonacci-like polynomials, generating function as denoted in Ref. 8, has the form:

$${℣}^{\left(x,y\right)}\left(v\right)=\sum {℣}_{n}\left(x,y\right)v^{n}=\frac{a+\left(b-\mathrm{ap}x\right)v}{a-pxv-\mathrm{qy}v^{2}}$$

The polynomials for the special cases can be obtained as presented in Table 1 for and of p,q,a,bandy .

**Table 1.** Values of Special cases of the generalized polynomial obtained by substituting specific values of the parameters. By choosing particular parameter combinations, the polynomial reduces to several well-known sequences such as the bivariate Fibonacci polynomials, Fibonacci polynomials, Pell polynomials, bivariate Lucas polynomials, Chebyshev polynomials of the second kind, and the Horadam polynomials.

(x,y)	(a,b)	(p,q)	${℣}_{n}\left(x,y\right)$
(x,y)	(0,1)	(1,1)	Bivariate Fibonacci ${℣}_{n}\left(x,y\right)$
(x,1)	(0,1)	(1,1)	Fibonacci ${℣}_{n}\left(x\right)$
(x,1)	(0,1)	(2,1)	Pell, $P_{n}\left(x\right)$
(x,y)	(2,x)	(1,1)	Bivariate Lucas $L_{n}\left(x,y\right)$
(t,−1)	(1,2t)	(2,1)	Chebyshev of the second kind $U_{n}\left(x\right)$
(x,1)	(a,bx)	(p,q)	Horadam $H_{n+1}\left(x\right)$

Consider ℓ to be the function of the form

$$\ell \left(v\right)=v+\sum_{m=2}^{\infty }\kappa_{m}v^{m},$$ (1.1)

that is part of Å where Å symbolizes the class of regular functions recognized on the disk

Ų={v∈ℂ:|v|<1},

regarding $\ell \left(0\right)=\ell^{´}\left(0\right)-1=0$ . Also, consider S to be the subclass of Å containing the form (1,1) . They are univalent in Ų . For each ℏ∈S , the image t=ℓ(v),v∈Ų is stated by The Koebe’s Covering Theorem. ^9^ In the t –plane, Ų, covers the disk {t:|t|<1/4} . Thus, every function from this theoremℏ∈S has an inverse $\hbar^{-1}$ , that holds

$$\ell^{-1}\left(\ell \left(v\right)\right)=v,\left(v\;\in \;Ų\right),$$

and,

$$\ell \left(\ell^{-1}\left(t\right)\right)=t,\left(\left|t\right|<r_{0}\left(\ell \right),r_{0}\left(\ell \right)\geq \frac{1}{4}\right),$$

where

$$ᶂ\left(t\right)=\ell^{-1}\left(t\right)=t-{ɛ}_{2}t^{2}+\left(2{ɛ}_{2}^{2}-{ɛ}_{3}\right)t^{3}-\left(5{ɛ}_{2}^{3}-5{ɛ}_{2}{ɛ}_{3}+{ɛ}_{4}\right)t^{4}+\cdot \cdot \cdot$$ (1.2)

In Ų, the function ℓ∈Å is named bi-univalent if both ℓ and $\ell^{-1}$ are univalent and the set of all bi-univalent functions are signified by Σ. Lewin ^10^ presented this set and revealed that $\left|{ɛ}_{2}\right|\leq 1.5$ regarding the function in the set Σ. Lately, Brannan and Clunie ^11^ estimated that $\left|{ɛ}_{2}\right|\leq \sqrt{2}$ and Netanyahu in ^12^ showed that $\;\left|{ɛ}_{2}\right|=\frac{4}{3}.$

Many authors recently offered and explored numerous remarkable subclasses of bi-univalent functions. ^2– 4, 13– 17^

The regular function ℓ is subordinate to ᶂ , written as

ℓ≺ᶂorℓ(v)≺ᶂ(v),(ѵ∈Ų), (1.3)

if there is regular function ƙ:Ų→Ų , with ƙ(0)=0 and |ƙ(v)|<1 such that

ℓ(v)=ᶂ(ƙ(v)),v∈Ų.

It follows the definition stating that:

ℓ(0)=ᶂ(0)andℓ(Ų)⊂ᶂ(Ų).

see Refs. 5, 6, 15, 18, 19.

Recent studies, including Refs. 16– 19, have emphasized the significant role of generalized polynomials and subordination techniques in obtaining coefficient estimates for various subclasses of bi-univalent functions, which further motivates the present investigation.

In the current study, a new subclass of bi-univalent functions filling the subordinate settings and it is defined by Fibonacci polynomial is offered. Moreover, the coefficient estimates for | ${ɛ}_{2}$ | and | ${ɛ}_{3}$ | are obtained for functions of the new classes.

## 2. Preliminaries

### The Family $M_{\Omega ,\Sigma }^{p,q,x,y}\left(X\left(ѵ\right)\right)$ and the Coefficients Bounds

**Definition 2.1:** A function ℓ∈∑ offered by (1.1) is stated to be in the set $M_{\Omega ,\Sigma }^{p,q,x,y}\left(X\left(ѵ\right)\right),0\;\leq \;\;\Omega \;\;\leq \;1,$ if it fits:

$$\left[\left(1-\Omega \right)\frac{\ell \left(z\right)}{z}+\Omega \frac{1+\frac{z\ell^{\prime \prime }\left(z\right)}{\ell^{\prime }\left(z\right)}}{\frac{z\ell^{\prime }\left(z\right)}{\ell \left(z\right)}}\right]≺\;X\left(ѵ\right)={℣}^{\left(x,y\right)}\left(ѵ\right)+1-a,\left(ѵ\;\in \;Ų\right)\;$$ (2.1)

$$\left[\left(1-\Omega \right)\frac{\ell \left(w\right)}{w}+\Omega \frac{1+\frac{w\ell^{\prime \prime }\left(w\right)}{\ell^{\prime }\left(w\right)}}{\frac{w\ell^{\prime }}{\ell \left(w\right)}}\right]≺\;X\left(t\right)={℣}^{\left(x,y\right)}\left(t\right)+1-a,\left(t\;\in \;Ų\right)◾$$ (2.2)

By assigning special values to parameters Ω and replacing polynomials, new and prominent families of bi-univalent functions are obtained. Accordingly, novel bounds for the initial coefficients are realized.

Remark 2.2: For Ω=1 , in $M_{\Omega ,\Sigma }^{p,q,x,y}\left(X\left(ѵ\right)\right)$ , we get the subfamilies ◾

Remark 2.3: Given a Horadam polynomial ${℣}^{\left(x,y\right)}$ with *b* = *b* x and *y* = 1, we obtain $M_{\Omega ,\Sigma }^{p,q,x,1}\left(X\left(ѵ\right)\right)$ . In this case for Ω=1 belongs to this family. Later, we have families $L_{\Sigma }\left(x\right)$ . ^7^

Remark 2.4: Consider ${℣}^{\left(x,y\right)}$ to be Chebyshev polynomials with *p* = 2, *q* = 1, a = 1, *b* = 2 *t*, x = *t*, *y* = -1. In Theorem 2.7 there is $\;M_{\Omega ,\Sigma }^{2,1,x,-1}\left(X\left(ѵ\right)\right).$ In such incident for Ω=0,1 belongs to this family. Then, the new *families* $M_{\Sigma }^{2,1,x,-1}\left(X\left(ѵ\right)\right)$ and $M_{1,\Sigma }^{2,1,x,-1}\left(X\left(ѵ\right)\right)\;$ respectively *are gained.*

Remark 2.5: Regard ${℣}^{\left(x,y\right)}$ as Fibonacci polynomials with *p* = *q* = 1, *b* = 1, *y* = 1, a = 0, there is. $\;M_{\Omega ,\Sigma }^{1,1,x,1}\left(X\left(ѵ\right)\right)$ . In this situation for Ω=0,1 be in this family. Then, new families $M_{\Sigma }^{1,1,x,1}\left(X\left(ѵ\right)\right)\;$ and $M_{1,\Sigma }^{1,1,x,1}\left(X\left(ѵ\right)\right)\;$ individually are got.

### The Family $\Gamma_{\Omega ,\Sigma }^{p,q,x,y}\left(X\left(ѵ\right)\right)$ and the Coefficients Bounds

Definition 2.6: For 0≤Ω≤1, a function ℓ∈∑ offered by (1.1) is stated to be in the set $\Gamma_{\Omega ,\Sigma }^{p,q,x,y}\left(X\left(ѵ\right)\right)$ if the following settings are satisfied:

$$\left[\left(1-\Omega \right)\ell^{\prime }\left(z\right)+\Omega \frac{1+\frac{z\ell^{\prime \prime }}{\ell^{\prime }}}{\frac{z\ell^{\prime }}{\ell \left(z\right)}}\right]≺X\left(ѵ\right)={℣}^{\left(x,y\right)}\left(ѵ\right)+1-a,\left(ѵ\;\in \;Ų\right)\;$$ (2.3)

$$\left[\left(1-\Omega \right)\ell^{\prime }\left(w\right)+\Omega \frac{1+\frac{w\ell^{\prime \prime }}{\ell^{\prime }}}{\frac{w\ell^{\prime }}{\ell \left(w\right)}}\right]≺X\left(t\right)={℣}^{\left(x,y\right)}\left(t\right)+1-a,\left(t\;\in \;Ų\right).$$ (2.4)

By assigning special values to parameters Ω and replacing polynomials, new and known families of bi-univalent functions are obtained. Thus, new bounds for the initial coefficients are gained.

Remark 2.7: For Ω=1 , in $\Gamma_{\Omega ,\Sigma }^{p,q,x,y}\left(X\left(ѵ\right)\right)$ , the subfamilies are got.

Remark 2.8: Consider ${℣}^{\left(x,y\right)}$ to be Horadam polynomial with *b = b* x and *y* = 1. Then, there is $\Gamma_{\Omega ,\Sigma }^{p,q,x,1}\left(X\left(ѵ\right)\right)\;$ . For Ω=1 be in this family. Then, families $L_{\Sigma }\left(x\right)$ ^7^ are got.

Remark 2.9: Let ${℣}^{\left(x,y\right)}$ is Chebyshev polynomials with *p* = 2, *b* = 2 *t* ,x = *t*, *y* = −1, *q* = 1, a = 1 in $\Gamma_{\Omega ,\Sigma }^{p,q,x,y}\left(X\left(ѵ\right)\right).\;\text{There is}$ $\;\Gamma_{\Omega ,\Sigma }^{2,1,x,-1}\left(X\left(ѵ\right)\right)$ . In such condition, for Ω=0,1 be in this family. Accordingly, new families $\Gamma_{\Sigma }^{2,1,x,-1}\left(X\left(ѵ\right)\right)$ and $\Gamma_{1,\Sigma }^{2,1,x,-1}\left(X\left(ѵ\right)\right)\;$ separately are obtained.

Remark 2.10: Let ${℣}^{\left(x,y\right)}$ is Fibonacci polynomials with *p* = *q* =1, *b* = 1, *y* = 1, a = 0, there is $\;\Gamma_{\Omega ,\Sigma }^{1,1,x,1}\left(X\left(ѵ\right)\right).$ In this case for Ω=0,1 be in this family. Then, new families $\Gamma_{\Sigma }^{1,1,x,1}\left(X\left(ѵ\right)\right)$ and $\Gamma_{1,\Sigma }^{1,1,x,1}\left(X\left(ѵ\right)\right)\;$ respectively are gained.

### Fekete-Szegö inequality for the class $M_{\Omega ,\Sigma }^{p,q,x,y}\left(X\left(ѵ\right)\right)$ and $\Gamma_{\Omega ,\Sigma }^{p,q,x,y}\left(X\left(ѵ\right)\right)$

Through applying the class $M_{\Omega ,\Sigma }^{p,q,x,y}\left(X\left(ѵ\right)\right)$ and $\Gamma_{\Omega ,\Sigma }^{p,q,x,y}\left(X\left(ѵ\right)\right)$ to the Fekete-Szegö problem, the following results and notes are realized:

Remark 2.11: Let ${℣}^{\left(x,y\right)}$ is Horadam polynomial with *b* = *b* x and *y* = 1, we obtain $M_{\Omega ,\Sigma }^{p,q,x,1}\left(X\left(ѵ\right)\right)$ . In this case for Ω=1 belongs to this family. Then, families $L_{\Sigma }\left(x\right)$ ^7^ are gained.

Remark 2.12: If *b* = *b* x and *y* = 1 and ${℣}^{\left(x,y\right)}$ is Horadam polynomial, we obtain $M_{,\Sigma }^{p,q,x,1}\left(X\left(ѵ\right)\right)$ .

Remark 2.13: Given a Horadam polynomial ${℣}^{\left(x,y\right)}$ with *b* = *b* x and *y* = 1, we obtain $\Gamma_{1,\Sigma }^{p,q,x,1}\left(X\left(ѵ\right)\right)$ . In this case for Ω=1 be in this family. Then, families $L_{\Sigma }\left(x\right)$ ^7^ are obtained.

Remark 2.14: Let ${℣}^{\left(x,y\right)}$ is Horadam polynomial with *b* = *bx* and *y* = 1, we have $\Gamma_{,\Sigma }^{p,q,x,1}\left(X\left(ѵ\right)\right)\;$ .

## 3. Main Results

Theorem 3.1: For, 0≤Ω≤1, let ℓ∈Σ given by (1.1) belong to $M_{\Omega ,\Sigma }^{p,q,x,y}\left(X\left(ѵ\right)\right)$ , then

$$\left|\kappa_{2}\right|\;\leq \;\frac{\left|b\right|\sqrt{\left|b\right|}}{\sqrt{\left|b^{2}\left(1-\Omega \right)-\left(\text{pbx}+\text{aqy}\right)\right|}}$$ (3.1)

$$\left|\kappa_{3}\right|\;\leq \;b^{2}+\frac{\left|b\right|}{\left(1+3\Omega \right)}◾$$ (3.2)

Proof: Since $\ell \;\in \;M_{\Omega ,\Sigma }^{p,q,x,y}\left(X\left(ѵ\right)\right)$ , there exist regular functions $i,\lambda :\hat{E}\;\to \;\hat{E}$ , such that

$$i\left(ѵ\right)=\sum_{1}^{\infty }i_{k}{ѵ}^{k},\lambda \left(t\right)=\sum_{1}^{\infty }\lambda_{k}t^{k}\;\text{and}\;\left|i_{k}\right|\;\leq \;1,\left|\lambda_{k}\right|\;\leq \;1,$$ (3.3)

we can write

$$\left[\left(1-\Omega \right)\frac{\ell \left(z\right)}{z}+\Omega \frac{1+\frac{z\ell^{\prime \prime }\left(z\right)}{\ell^{\prime }\left(z\right)}}{\frac{z\ell^{\prime }\left(z\right)}{\ell \left(z\right)}}\right]=X\left(ѵ\right),\left(ѵ\;\in \;Ų\right)$$ (3.4)

$$\left[\left(1-\Omega \right)\frac{\ell \left(w\right)}{w}+\Omega \frac{1+\frac{w\ell^{\prime \prime }\left(w\right)}{\ell^{\prime }\left(w\right)}}{\frac{w\ell^{\prime }}{\ell \left(w\right)}}\right]=X\left(t\right),\left(t\;\in \;Ų\right)◾$$ (3.5)

Equivalently,

$$1+\kappa_{2}z+\left\{\left(1+3\Omega \right)\kappa_{3}-4\Omega {\kappa_{2}}^{2}\right\}z^{2}+...=1+{℣}_{1}\left(x,y\right)i_{1}z+\left({℣}_{1}\left(x,y\right)i_{2}+{℣}_{2}\left(x,y\right){i_{1}}^{2}\right)z^{2}+\ldots$$ (3.6)

and

$$1-\kappa_{2}w+\left\{2\left(1+\Omega \right){\kappa_{2}}^{2}-\left(1+3\Omega \right)\kappa_{3}\right\}w^{2}-...=1+{℣}_{1}\left(x,y\right)\lambda_{1}w+\left({℣}_{1}\left(x,y\right)\lambda_{2}+{℣}_{2}\left(x,y\right){\lambda_{1}}^{2}\;\right)w^{2}+\ldots$$ (3.7)

Thus upon comparing the corresponding coefficients in (3.6) and (3.7), we have

$$\kappa_{2}={℣}_{1}\left(x,y\right)i_{1}$$ (3.8)

$$\left(1+3\Omega \right)\kappa_{3}-4\Omega {\kappa_{2}}^{2}={℣}_{1}\left(x,y\right)i_{2}+{℣}_{2}\left(x,y\right){i_{1}}^{2}$$ (3.9)

$$-\kappa_{2}={℣}_{1}\left(x,y\right)\lambda_{1}\;$$ (3.10)

and

$$2\left(1+\Omega \right){\kappa_{2}}^{2}-\left(1+3\Omega \right)\kappa_{3}={℣}_{1}\left(x,y\right)\lambda_{2}+{℣}_{2}\left(x,y\right){\lambda_{1}}^{2}◾$$ (3.11)

Now considering (3.8) and (3.10), we get

$$i_{1}=-\lambda_{1}$$ (3.12)

$$\begin{array}{c} 2{\kappa_{2}}^{2}={{℣}^{2}}_{1}\left(x,y\right)\left({i_{1}}^{2}+{\lambda_{1}}^{2}\right)\\ {\kappa_{2}}^{2}=\frac{{{℣}^{2}}_{1}\left(x,y\right)\left({i_{1}}^{2}+{\lambda_{1}}^{2}\right)}{2}◾ \end{array}$$ (3.13)

If we add (3.9) to (3.11), we find that

$$2\left(1-\Omega \right){\kappa_{2}}^{2}={℣}_{1}\left(x,y\right)\left(i_{2}+\lambda_{2}\right)+{℣}_{2}\left(x,y\right)\left({i_{1}}^{2}+{\lambda_{1}}^{2}\right)$$ (3.14)

Upon substituting the value of ${i_{1}}^{2}+{\lambda_{1}}^{2}$ from (3.13) in (3.14), we deduce the next result:

$$2\left(1-\Omega \right){\kappa_{2}}^{2}={℣}_{1}\left(x,y\right)\left(i_{2}+\lambda_{2}\right)+{℣}_{2}\left(x,y\right)\frac{2{\kappa_{2}}^{2}}{{{℣}^{2}}_{1}\left(x,y\right)}$$

$$2{{℣}^{2}}_{1}\left(x,y\right)\left(1-\Omega \right){\kappa_{2}}^{2}={{℣}^{3}}_{1}\left(x,y\right)\left(i_{2}+\lambda_{2}\right)+{℣}_{2}\left(x,y\right)2{\kappa_{2}}^{2}◾$$

And more simplify

$$2\left({{℣}^{2}}_{1}\left(x,y\right)\left(1-\Omega \right)-{℣}_{2}\left(x,y\right)\right){\kappa_{2}}^{2}={{℣}^{3}}_{1}\left(x,y\right)\left(i_{2}+\lambda_{2}\right)$$

$${\kappa_{2}}^{2}=\frac{{{℣}^{3}}_{1}\left(x,y\right)\left(i_{2}+\lambda_{2}\right)}{2\left({{℣}^{2}}_{1}\left(x,y\right)\left(1-\Omega \right)-{℣}_{2}\left(x,y\right)\right)}$$

$${\left|\kappa_{2}\right|}^{2}\;\leq \;\frac{\left|{{℣}^{3}}_{1}\left(x,y\right)\right|}{\left|{{℣}^{2}}_{1}\left(x,y\right)\left(1-\Omega \right)-{℣}_{2}\left(x,y\right)\right|}$$

Using the inequality $\left|i_{k}\right|\;\leq \;1,\left|\lambda_{k}\right|\;\leq \;1$ ◾

$$\left|\kappa_{2}\right|\;\leq \;\frac{\left|b\right|\sqrt{\left|b\right|}}{\sqrt{\left|b^{2}\left(1-\Omega \right)-\left(\text{pbx}+\text{aqy}\right)\right|}}.$$ (3.15)

Subtracting (3.9) and (3.11), and with some computation, we have

$$2\left(1+3\Omega \right)\kappa_{3}-2\left(1+3\Omega \right){\kappa_{2}}^{2}={℣}_{1}\left(x,y\right)\left(i_{2}-\lambda_{2}\right)$$ (3.16)

Put (3.13) in (3.16), then we deduce

$$\begin{array}{c} \left|\kappa_{3}\right|\;\leq \;\frac{\left|{{℣}^{2}}_{1}\left(x,y\right)\left({i_{1}}^{2}+{\lambda_{1}}^{2}\right)\right|}{2}+\frac{\left|{℣}_{1}\left(x,y\right)\left(i_{2}-\lambda_{2}\right)\right|}{2\left(1+3\Omega \right)}.\\ \left|\kappa_{3}\right|\;\leq \;b^{2}+\frac{\left|b\right|}{\left(1+3\Omega \right)}◾ \end{array}$$ (3.17)

Corollary 3.2:

(i) Consider ℓ presented by (1.1) be in the subfamily $M_{1,\Sigma }^{p,q,x,y}\left(X\left(ѵ\right)\right)$ . Then,
$$\left|\kappa_{2}\right|\;\leq \;\frac{\left|b\right|\sqrt{\left|b\right|}}{\sqrt{\left|\mathrm{pb}x+\text{aqy}\right|}}.$$

$$\left|\kappa_{3}\right|\;\leq \;b^{2}+\frac{\left|b\right|}{4}◾$$

(ii) Let ℓ offered by (1.1) be in the sub family $M_{\Sigma }^{p,q,x,y}\left(X\left(ѵ\right)\right)$ . Then,
$$\left|\kappa_{2}\right|\;\leq \;\frac{\left|b\right|\sqrt{\left|b\right|}}{\sqrt{\left|b^{2}-\left(\text{pbx}+\text{aqy}\right)\right|}}.$$

$$\left|\kappa_{3}\right|\;\leq \;b^{2}+\left|b\right|∎$$

Corollary 3.3:

(i) Regard ℓ introduced by (1.1) be in the subfamily $M_{\Omega ,\Sigma }^{p,q,x,1}\left(X\left(ѵ\right)\right)$ and ℣(x,y) be Horadam $H_{n+1}\left(x\right)$ . Then,
$$\left|\kappa_{2}\right|\;\leq \;\frac{\left|\mathrm{bx}\right|\sqrt{\left|bx\right|}}{\sqrt{\left|b^{2}x^{2}\left(1-\Omega \right)-\left(\text{pb}x^{2}+\mathrm{aq}\right)\right|}}.$$
$$\left|\kappa_{3}\right|\;\leq \;b^{2}x^{2}+\frac{\left|\mathrm{bx}\right|}{\left(1+3\Omega \right)}◾$$
(ii) Let ℓ offered by (1.1) and Ω=1 be in the sub family $M_{,\Sigma }^{p,q,x,1}\left(X\left(ѵ\right)\right)$ . Then,
$$\left|\kappa_{2}\right|\;\leq \;\frac{\left|\mathrm{bx}\right|\sqrt{\left|bx\right|}}{\sqrt{\left|b^{2}x^{2}-\left(\text{pb}x^{2}+\mathrm{aq}\right)\right|}}.$$
$$\left|\kappa_{3}\right|\;\leq \;b^{2}x^{2}+\left|bx\right|◾$$

Corollary 3.4:

(i) Let ℓ∈Σ shown by (1.1) in the subfamily $M_{\Omega ,\Sigma }^{2,1,x,-1}\left(X\left(ѵ\right)\right)$ and ${℣}^{\left(x,y\right)}$ be Chebyshev polynomials with *p* = 2, *b* = 2 *t*, x = *t*, *y* = −1, *q* = 1, and a = 1. Then,
$$\left|\kappa_{2}\right|\;\leq \;\frac{\left|2t\right|\sqrt{\left|2t\right|}}{\sqrt{\left|4t^{4}\left(1-\Omega \right)-\left(4t^{3}-1\right)\right|}}.$$
$$\left|\kappa_{3}\right|\;\leq \;4t^{4}+\frac{2\left|t^{2}\right|}{\left(1+3\Omega \right)}◾$$
(ii) Consider ℓ∈Σ offered by (1.1) in the subfamily $M_{,\Sigma }^{2,1x,-1}\left(X\left(ѵ\right)\right)$ . Then,
$$\left|\kappa_{2}\right|\;\leq \;\frac{\left|2t\right|\sqrt{\left|2t\right|}}{\sqrt{\left|4t^{4}-\left(4t^{3}-1\right)\right|}}.$$
$$\left|\kappa_{3}\right|\;\leq \;4t^{4}+2\left|t^{2}\right|◾$$
(iii) Let ℓ∈Σ presented by (1.1) in the subfamily $M_{1,\Sigma }^{2,1,x,-1}\left(X\left(ѵ\right)\right)$ . Then,
$$\left|\kappa_{2}\right|\;\leq \;\frac{\left|2t\right|\sqrt{\left|2t\right|}}{\sqrt{\left|(4t^{3}-1)\right|}}.$$
$$\left|\kappa_{3}\right|\;\leq \;4t^{4}+\frac{\left|t^{2}\right|}{2}◾$$

Corollary 3.5:

(i) Consider ℓ∈Σ stated by (1.1) in the subfamily $M_{\Omega ,\Sigma }^{1,1,x,1}\left(X\left(ѵ\right)\right)$ and ${℣}^{\left(x,y\right)}$ be Fibonacci polynomials with *p* = 1, *q* = 1, *b* = 1, *y* = 1, and a = 0. Then,
$$\left|\kappa_{2}\right|\;\leq \;\frac{1}{\sqrt{\left|\left(1-\Omega \right)-x\right|}}.$$
$$\left|\kappa_{3}\right|\;\leq \;1+\frac{1}{\left(1+3\Omega \right)}◾$$
(ii) Let ℓ∈Σ revealed by (1.1) in the subfamily $M_{,\Sigma }^{1,1x,1}\left(X\left(ѵ\right)\right)$ . Then,
$$\left|\kappa_{2}\right|\;\leq \;\frac{1}{\sqrt{\left|1-x\right|}}.$$
$$\left|\kappa_{3}\right|\;\leq \;2◾$$
(iii) Let ℓ∈Σ stated by (1.1) in the set $M_{1,\Sigma }^{1,1,x,1}\left(X\left(ѵ\right)\right)$
$$\left|\kappa_{2}\right|\;\leq \;\frac{1}{\sqrt{\left|x\right|}}.$$
$$\left|\kappa_{3}\right|\;\leq \;\frac{5}{4}◾$$

Theorem 3.6: For 0≤Ω≤1, let ℓ∈Σ offered by (1.1) belong to $\;\Gamma_{\Omega ,\Sigma }^{p,q,x,y}\left(X\left(ѵ\right)\right)$ . Then,

$$\;\left|\kappa_{2}\right|\;\leq \;\frac{\left|b\right|\sqrt{\left|b\right|}}{\sqrt{\left|3\left(1-\Omega \right)b^{2}-{\left(2-\Omega \right)}^{2}\left(\mathrm{pbx}+\text{aqy}\right)\right|}}.$$ (3.18)

$$\left|\kappa_{3}\right|\;\leq \;\frac{\left(3-\Omega \right)b^{2}}{\left(3+\Omega \right){\left(2-\Omega \right)}^{2}}+\frac{\left|b\right|}{\left(3+\Omega \right)}.$$ (3.19)

Proof: As $\ell \;\in \;\Gamma_{\Omega ,\Sigma }^{p,q,x,y}\left(X\left(ѵ\right)\right)$ , there exists systematic functions $\;i,\lambda :\hat{E}\;\to \;\hat{E}$ , such that

$$i\left(ѵ\right)=\sum_{1}^{\infty }i_{k}{ѵ}^{k},\lambda \left(t\right)=\sum_{1}^{\infty }\lambda_{k}t^{k}\;\text{and}\;\left|i_{k}\right|\;\leq \;1,\left|\lambda_{k}\right|\;\leq \;1,$$ (3.20)

It is possible to write:

$$\left[\left(1-\Omega \right)\ell^{\prime }\left(z\right)+\Omega \frac{1+\frac{z\ell^{\prime \prime }\left(z\right)}{\ell^{\prime }\left(z\right)}}{\frac{z\ell^{\prime }\left(z\right)}{\ell \left(z\right)}}\right]=X\left(ѵ\right),\left(ѵ\;\in \;Ų\right)\;$$

$$\left[\left(1-\Omega \right)\ell^{\prime }\left(w\right)+\Omega \frac{1+\frac{w\ell^{\prime \prime }\left(w\right)}{\ell^{\prime }\left(w\right)}}{\frac{w\ell^{\prime }}{\ell \left(w\right)}}\right]=X\left(t\right),\left(t\;\in \;Ų\right)$$

$$1+\left(2-\Omega \right)\kappa_{2}z+\left[\left(3+\Omega \right)\kappa_{3}-4\Omega {\kappa_{2}}^{2}\right]z^{2}\ldots .=1+{℣}_{1}\left(x,y\right){ɍ}_{1}z+\left({℣}_{1}\left(x,y\right){ɍ}_{2}+{℣}_{2}\left(x,y\right){{ɍ}_{1}}^{2}\right)z^{2}+\ldots$$ (3.21)

and

$$1-\left(2-\Omega \right)\kappa_{2}w+\left[\left(6-2\Omega \right){\kappa_{2}}^{2}-\left(3+\Omega \right)\kappa_{3}\right]w^{2}...=1+{℣}_{1}\left(x,y\right){ѳ}_{1}w+\left({℣}_{1}\left(x,y\right){ѳ}_{2}+{℣}_{2}\left(x,y\right){{ѳ}_{1}}^{2}\;\right)w^{2}+\ldots$$ (3.22)

Thus upon comparing the corresponding coefficients in (3.21) and (3.22), we have

$$\left(2-\Omega \right)\kappa_{2}={℣}_{1}\left(x,y\right){ɍ}_{1}$$ (3.23)

$$\left(3+\Omega \right)\kappa_{3}-4\Omega {\kappa_{2}}^{2}={℣}_{1}\left(x,y\right){ɍ}_{2}+{℣}_{2}\left(x,y\right){{ɍ}_{1}}^{2}$$ (3.24)

$$-\left(2-\Omega \right)\kappa_{2}={℣}_{1}\left(x,y\right){ѳ}_{1}$$ (3.25)

$$-\left[\left(3+\Omega \right)\kappa_{3}-\left(6-2\Omega \right){\kappa_{2}}^{2}\right]={℣}_{1}\left(x,y\right){ѳ}_{2}+{℣}_{2}\left(x,y\right){{ѳ}_{1}}^{2}$$ (3.26)

Now considering (3.23) and (3.25), we get

$${ɍ}_{1}=-{ѳ}_{1}$$ (3.27)

$$\begin{array}{c} 2{\left(2-\Omega \right)}^{2}{\kappa_{2}}^{2}={{℣}^{2}}_{1}\left(x,y\right)\left({{ɍ}_{1}}^{2}+{{ѳ}_{1}}^{2}\right)\\ {\kappa_{2}}^{2}=\frac{{{℣}^{2}}_{1}\left(x,y\right)\left({{ɍ}_{1}}^{2}+{{ѳ}_{1}}^{2}\right)}{2{\left(2-\Omega \right)}^{2}} \end{array}$$ (3.28)

In adding (3.24) to (3.26), we obtain:

$$\left[6\left(1-\Omega \right){{℣}^{2}}_{1}\left(x,y\right)-2{℣}_{2}\left(x,y\right){\left(2-\Omega \right)}^{2}\right]{\kappa_{2}}^{2}={{℣}^{3}}_{1}\left(x,y\right)\left({ɍ}_{2}+{ѳ}_{2}\right)$$

$${\kappa_{2}}^{2}=\frac{{{℣}^{3}}_{1}\left(x,y\right)\left({ɍ}_{2}+{ѳ}_{2}\right)}{6\left(1-\Omega \right){{℣}^{2}}_{1}\left(x,y\right)-2{℣}_{2}\left(x,y\right){\left(2-\Omega \right)}^{2}}$$

$${\left|\kappa_{2}\right|}^{2}\;\leq \;\frac{\left|{{℣}^{3}}_{1}\left(x,y\right)\right|}{\left|3\left(1-\Omega \right){{℣}^{2}}_{1}\left(x,y\right)-{℣}_{2}\left(x,y\right){\left(2-\Omega \right)}^{2}\right|}$$

$$\left|\kappa_{2}\right|\;\leq \;\frac{\left|b\right|\sqrt{\left|b\right|}}{\sqrt{\left|3\left(1-\Omega \right)b^{2}-{\left(2-\Omega \right)}^{2}\left(\mathrm{pbx}+\text{aqy}\right)\right|}}.$$

Subtracting (3.24) and (3.26), and with some computation, we get

$$\begin{array}{c} \left(3+\Omega \right)\kappa_{3}-4\Omega {\kappa_{2}}^{2}={℣}_{1}\left(x,y\right){ɍ}_{2}+{℣}_{2}\left(x,y\right){{ɍ}_{1}}^{2}\\ -\left[\left(3+\Omega \right)\kappa_{3}-\left(6-2\Omega \right){\kappa_{2}}^{2}\right]={℣}_{1}\left(x,y\right){ѳ}_{2}+{℣}_{2}\left(x,y\right){{ѳ}_{1}}^{2}\\ 2\left(3+\Omega \right)\kappa_{3}-\left(6+2\Omega \right){\kappa_{2}}^{2}={℣}_{1}\left(x,y\right)\left({ɍ}_{2}-{ѳ}_{2}\right) \end{array}$$ (3.29)

Put (3.28) in (3.29)

$$2\left(3+\Omega \right)\kappa_{3}-\left(6+2\Omega \right)\frac{{{℣}^{2}}_{1}\left(x,y\right)\left({{ɍ}_{1}}^{2}+{{ѳ}_{1}}^{2}\right)}{2{\left(2-\Omega \right)}^{2}}={℣}_{1}\left(x,y\right)\left({ɍ}_{2}-{ѳ}_{2}\right)$$

$$4\left(3+\Omega \right){\left(2-\Omega \right)}^{2}\kappa_{3}-\left(6-2\Omega \right){{℣}^{2}}_{1}\left(x,y\right)\left({{ɍ}_{1}}^{2}+{{ѳ}_{1}}^{2}\right)=2{\left(2-\Omega \right)}^{2}{℣}_{1}\left(x,y\right)\left({ɍ}_{2}-{ѳ}_{2}\right)$$

$$\kappa_{3}=\frac{\left(6-2\Omega \right){{℣}^{2}}_{1}\left(x,y\right)\left({{ɍ}_{1}}^{2}+{{ѳ}_{1}}^{2}\right)}{4\left(3+\Omega \right){\left(2-\Omega \right)}^{2}}+\frac{2{\left(2-\Omega \right)}^{2}{℣}_{1}\left(x,y\right)\left({ɍ}_{2}-{ѳ}_{2}\right)}{4\left(3+\Omega \right){\left(2-\Omega \right)}^{2}}$$

$$\left|\kappa_{3}\right|\;\leq \;\frac{\left(3-\Omega \right)b^{2}}{\left(3+\Omega \right){\left(2-\Omega \right)}^{2}}+\frac{\left|b\right|}{\left(3+\Omega \right)}◾$$

Corollary 3.7:

(i) Let ℓ stated by (1.1) be in the subfamily $\Gamma_{\Sigma }^{p,q,x,y}\left(X\left(ѵ\right)\right)$ . Then,
$$\left|\kappa_{2}\right|\;\leq \;\frac{\left|b\right|\sqrt{\left|b\right|}}{\sqrt{\left|3b^{2}-4\left(\mathrm{pbx}+\text{aqy}\right)\right|}}.$$
$$\left|\kappa_{3}\right|\;\leq \;\frac{\;b^{2}}{4}+\frac{\left|b\right|}{3}◾$$
(ii) Let ℓ given by (1.1) be in the subfamily $\Gamma_{1,\Sigma }^{p,q,x,y}\left(X\left(ѵ\right)\right)$ . Then,
$$\left|\kappa_{2}\right|\;\leq \;\frac{\left|b\right|\sqrt{\left|b\right|}}{\sqrt{\left|(\mathrm{pb}x+\text{aqy})\right|}}.$$
$$\left|\kappa_{3}\right|\;\leq \;\frac{\left|b\right|}{4\;}◾$$

Corallary 3.8: If ${℣}_{n}$ is Horadam polynomial in above theorem Then, we have

$$\begin{array}{c} \left|\kappa_{2}\right|\;\leq \;\frac{\left|\mathrm{bx}\right|\sqrt{\left|\text{bx}\right|}}{\sqrt{\left|3\left(1-\Omega \right){\left(\text{bx}\right)}^{2}-{\left(2-\Omega \right)}^{2}\left(\mathrm{pb}x^{2}+\mathrm{aq}\right)\right|}},\\ \left|\kappa_{3}\right|\;\leq \;\frac{3\left(1-\Omega \right){\left(\text{bx}\right)}^{2}}{\left(3+\Omega \right){\left(2-\Omega \right)}^{2}}+\frac{\left|\text{bx}\right|}{\left(3+\Omega \right)}◾ \end{array}$$

Corallary 3.9: Where ${℣}_{n}\left(x,y\right)=px{℣}_{n-1}\left(x,y\right)-\mathrm{qy}{℣}_{n-2}\left(x,y\right),n\;\geq \;2\;$ ,

$${℣}_{0}\left(x,y\right)=a,{℣}_{1}\left(x,y\right)=b\;$$

If ${℣}_{n}$ is generalized bivariate Fibonacci-like polynomials and Ω = 0. Then,

$$\left|\kappa_{2}\right|\;\leq \;\frac{\left|b\right|\sqrt{\left|b\right|}}{\sqrt{3b^{2}-4\left(\text{pbx}-\text{aqy}\right)\;}}.$$

Corallary 3.10: If ${℣}_{n}$ is Horadam polynomial and Ω = 0 (remark)

$${H^{3}}_{1}\left(x,y\right)={\left(\mathrm{bx}\right)}^{3},H_{2}\left(x,y\right)=\text{pb}x^{2}-\mathrm{aq}$$

$$\left|\kappa_{2}\right|\;\leq \;\frac{\left|\mathrm{bx}\right|\sqrt{\left|\text{bx}\right|}}{\sqrt{3b^{2}x^{2}-4\left(\text{pb}x^{2}-\mathrm{aq}\right)\;}}.$$

$$\left|\kappa_{3}\right|\;\leq \;\frac{{\left(\text{bx}\right)}^{2}}{4}+\frac{\left|\text{bx}\right|}{3}◾$$

Theorem 3.11: Suppose $\ell \left(ѵ\right)=ѵ+\sum_{m=2}^{\infty }{ɛ}_{m}{ѵ}^{m}\;\in \;M_{\Omega ,\Sigma }^{p,q,x,y}\left(X\left(ѵ\right)\right)$ , then,

$$|\kappa_{3}-\nu \;{\kappa_{2}}^{2}\;|=\;\left\{ \begin{array}{lll} \frac{\left|b\right|}{\left(1+3\Omega \right)} & \text{if} & \left|1-V\right|\;\leq \;\frac{\left|\left(1-\Omega \right)b^{2}-\left(\mathrm{pbx}+\text{aqy}\right)\right|}{\left(1+3\Omega \right)b^{2}}\\ \frac{\left(1-V\right){\left|b\right|}^{3}}{\left|(1-\Omega \right)b^{2}-\left(\mathrm{pbx}+a\mathrm{qy})\right|} & \text{if} & \left|1-V\right|>\frac{\left|\left(1-\Omega \right)b^{2}-\left(\mathrm{pbx}+\text{aqy}\right)\right|}{\left(1+3\Omega \right)b^{2}} \end{array}\right.$$

Proof:
$$\kappa_{3}-\nu {\kappa_{2}}^{2}=\frac{{℣}_{1}\left(x,y\right)\left({ɍ}_{2}-{ѳ}_{2}\right)}{2\left(1+3\Omega \right)}+{\kappa_{2}}^{2}-\nu {\kappa_{2}}^{2}$$

$$\kappa_{3}-{{\nu \kappa }_{2}}^{2}=\frac{b\left({ɍ}_{2}-{ѳ}_{2}\right)}{2\left(1+3\Omega \right)}+\frac{b^{3}\left({ɍ}_{2}+{ѳ}_{2}\right)}{2\left(b^{2}\left(1-\Omega \right)-\left(\mathrm{pbx}+\text{aqy}\right)\right)}\left(1-\nu \right)$$

$$\kappa_{3}-{{\nu \kappa }_{2}}^{2}=\left\{\frac{\left(1-\nu \right)b^{3}}{2\left(b^{2}\left(1-\Omega \right)-\left(\mathrm{pbx}+\text{aqy}\right)\right)}+\frac{b}{2\left(1+3\Omega \right)}\right\}{ɍ}_{2}+\left\{\frac{\left(1-\nu \right)b^{3}}{2\left(b^{2}\left(1-\Omega \right)-\left(\mathrm{pbx}+\text{aqy}\right)\right)}-\frac{b}{2\left(1+3\Omega \right)}\right\}{ѳ}_{2}$$

$$\kappa_{3}-{{\nu \kappa }_{2}}^{2}=\left\{h\left(\nu ,\Omega \right)+\frac{b}{2\left(1+3\Omega \right)}\right\}{ɍ}_{2}+\left\{h\left(\nu ,\Omega \right)-\frac{b}{2\left(1+3\Omega \right)}\right\}{ѳ}_{2}$$

Where

$$h\left(\nu ,\Omega \right)=\frac{\left(1-\nu \right)b^{3}}{2\left(b^{2}\left(1-\Omega \right)-\left(\mathrm{pbx}+\text{aqy}\right)\right)}$$

$$\kappa_{3}-{{\nu \kappa }_{2}}^{2}=\left(h\left(\nu \right)+\frac{b}{2\left(3+\Omega \right)}\right){ɍ}_{2}+\left(h\left(\nu \right)-\frac{b}{2\left(3+\Omega \right)}\right){ѳ}_{2}$$

Corollary 3.12: Let ℓ assumed by (1.1) be in the subfamily $M_{1,\Sigma }^{p,q,x,1}\left(X\left(ѵ\right)\right)$ and ℣(x,y) be Horadam $H_{n+1}\left(x\right)$ . Then,

$$|\kappa_{3}-\nu \;{\kappa_{2}}^{2}\;|=\;\left\{ \begin{array}{lll} \frac{\left|\text{bx}\right|}{4} & \text{if} & \left|1-V\right|\;\leq \;\frac{\left|p{\text{bx}}^{2}+\mathrm{aq}\right|}{4{\left(\text{bx}\right)}^{2}}\\ \frac{\left(1-V\right){\left|\text{bx}\right|}^{3}}{\left|p{\text{bx}}^{2}+\mathrm{aq}\right|} & \text{if} & \left|1-V\right|>\frac{\left|p{\text{bx}}^{2}+\mathrm{aq}\right|}{4{\left(\text{bx}\right)}^{2}} \end{array}\right.$$

Corollary 3.13: Let ℓ offered by (1.1) be in the subfamily $M_{,\Sigma }^{p,q,x,1}\left(X\left(ѵ\right)\right)$ and ℣(x,y) be Horadam $H_{n+1}\left(x\right)$ . Then,

$$|\kappa_{3}-\nu \;{\kappa_{2}}^{2}\;|=\;\left\{ \begin{array}{lll} \left|bx\right| & \text{if} & \left|1-V\right|\;\leq \;\frac{\left|{\left(bx\right)}^{2}-\left(\mathrm{pb}x^{2}+\mathrm{aq}\right)\right|}{{\left(bx\right)}^{2}}\\ \frac{\left(1-V\right){\left|bx\right|}^{3}}{\left|{\left(bx\right)}^{2}-\left(\mathrm{pb}x^{2}+\mathrm{aq}\right)\right|} & \text{if} & \left|1-V\right|>\frac{\left|{\left(bx\right)}^{2}-\left(\mathrm{pb}x^{2}+\mathrm{aq}\right)\right|}{{\left(bx\right)}^{2}} \end{array}\right.$$

Theorem 3.14: Suppose that $\ell \left(ѵ\right)=ѵ+\sum_{m=2}^{\infty }{ɛ}_{k}{ѵ}^{m}\;\in \;\Gamma_{\Omega ,\Sigma }^{p,q,x,y}\left(X\left(ѵ\right)\right)$ . Then

$$|\kappa_{3}-\nu \;{\kappa_{2}}^{2}\;|=\left\{ \begin{array}{lll} \frac{\left|b\right|}{\left(3+\Omega \right)} & \text{if} & \left|1-V\right|\;\leq \;\frac{\left|3\left(1-\Omega \right)b^{2}-\left(\mathrm{pbx}+\text{aqy}\right){\left(2-\Omega \right)}^{2}\right|}{\left(3+\Omega \right)b^{2}}\\ \frac{\left(1-V\right){\left|b\right|}^{3}}{\left|3\left(1+\Omega \right)b^{2}-\left(\text{pbx}+\text{aqy}\right){\left(2-\Omega \right)}^{2}\right|} & \text{if} & \left|1-V\right|>\frac{\left|3\left(1-\Omega \right)b^{2}-\left(\mathrm{pbx}+\text{aqy}\right){\left(2-\Omega \right)}^{2}\right|}{\left(3+\Omega \right)b^{2}} \end{array}\right.$$

Proof:
$$\kappa_{3}-{{\mathrm{v\kappa}}_{2}}^{2}=\frac{{℣}_{1}\left(x,y\right)\left({ɍ}_{2}-{ѳ}_{2}\right)}{2\left(3+\Omega \right)}+\frac{\left(6-2\Omega \right)}{2\left(3+\Omega \right)}{\kappa_{2}}^{2}-\nu {\kappa_{2}}^{2}$$

$$\kappa_{3}-{{\mathrm{v\kappa}}_{2}}^{2}=\frac{{℣}_{1}\left(x,y\right)\left({ɍ}_{2}-{ѳ}_{2}\right)}{2\left(3+\Omega \right)}+\left(1-\nu \right)\frac{{{℣}^{3}}_{1}\left(x,y\right)\left({ɍ}_{2}+{ѳ}_{2}\right)}{6\left(1-\Omega \right){{℣}^{2}}_{1}\left(x,y\right)-2{℣}_{2}\left(x,y\right){\left(2-\Omega \right)}^{2}}$$

$$\kappa_{3}-{{\mathrm{v\kappa}}_{2}}^{2}=\left(\frac{\left(1-\nu \right)b^{3}}{6\left(1-\Omega \right)b^{2}-2\left(\text{pbx}+\text{aqy}\right){\left(2-\Omega \right)}^{2}}+\frac{b}{2\left(3+\Omega \right)}\right){ɍ}_{2}+\left(\frac{\left(1-\nu \right)b^{3}}{6\left(1-\Omega \right)b^{2}-2\left(\text{pbx}+\text{aqy}\right){\left(2-\Omega \right)}^{2}}-\frac{b}{2\left(3+\Omega \right)}\right){ѳ}_{2}$$

put

$$h\left(\nu \right)=\frac{\left(1-\nu \right)b^{3}}{6\left(1-\Omega \right)b^{2}-2\left(\text{pbx}+\text{aqy}\right){\left(2-\Omega \right)}^{2}}$$

$$\kappa_{3}-{{\mathrm{v\kappa}}_{2}}^{2}=\left(h\left(\nu \right)+\frac{b}{2\left(3+\Omega \right)}\right){ɍ}_{2}+\left(h\left(\nu \right)-\frac{b}{2\left(3+\Omega \right)}\right){ѳ}_{2}$$

$$|\kappa_{3}-\nu \;{\kappa_{2}}^{2}\;|=\left\{ \begin{array}{lll} \frac{\left|b\right|}{\left(3+\Omega \right)} & \text{if} & \left|1-V\right|\;\leq \;\frac{\left|3\left(1-\Omega \right)b^{2}-\left(\mathrm{pbx}+\text{aqy}\right){\left(2-\Omega \right)}^{2}\right|}{\left(3+\Omega \right)b^{2}}\\ \frac{\left(1-V\right){\left|b\right|}^{3}}{\left|3\left(1+\Omega \right)b^{2}-\left(\text{pbx}+\text{aqy}\right){\left(2-\Omega \right)}^{2}\right|} & \text{if} & \left|1-V\right|>\frac{\left|3\left(1-\Omega \right)b^{2}-\left(\mathrm{pbx}+\text{aqy}\right){\left(2-\Omega \right)}^{2}\right|}{\left(3+\Omega \right)b^{2}} \end{array}\right.$$

Corollary 3.15: Let ℓ offered by (1.1) be in the subfamily $\Gamma_{1,\Sigma }^{p,q,x,1}\left(X\left(ѵ\right)\right)\;$ and ℣(x,y) be Horadam $H_{n+1}\left(x\right)$ . Then

$$|\kappa_{3}-\nu \;{\kappa_{2}}^{2}\;|=\;\left\{ \begin{array}{lll} \frac{\left|bx\right|}{3} & \text{if} & \left|1-V\right|\;\leq \;\frac{\left|3{\text{bx}}^{2}-4\left(\mathrm{pb}x^{2}+\mathrm{aq}\right)\right|}{3{\left(\text{bx}\right)}^{2}}\\ \frac{\left(1-V\right){\left|b\right|}^{3}}{\left|3{\left(bx\right)}^{2}-4\left(\text{pb}x^{2}+aq\right)\right|} & \text{if} & \left|1-V\right|>\frac{\left|3{bx}^{2}-4\left(\mathrm{pb}x^{2}+\mathrm{aq}\right)\right|}{3{\left(bx\right)}^{2}} \end{array}\right.$$

Corollary 3.16: Let ℓ offered by (1.1) be in the subfamily $\;\Gamma_{,\Sigma }^{p,q,x,1}\left(X\left(ѵ\right)\right)\;$ and ℣(x,y) be Horadam $H_{n+1}\left(x\right)$ . Then

$$|\kappa_{3}-\nu \;{\kappa_{2}}^{2}\;|=\;\left\{ \begin{array}{lll} \frac{\left|bx\right|}{4} & \text{if} & \left|1-V\right|\;\leq \;\frac{\left|(\mathrm{pb}x^{2}+\mathrm{aq})\right|}{4b^{2}}\\ \frac{\left(1-V\right){\left|b\right|}^{3}}{\left|6b^{2}-\left(\text{pb}x^{2}+aq\right)\right|} & \text{if} & \left|1-V\right|>\frac{\left|(\mathrm{pb}x^{2}+\mathrm{aq})\right|}{4b^{2}} \end{array}\right.$$

## Ethical considerations

This study was not conducted on human or animal participants; therefore this field was not required.

## Data Availability

No data associated with this article. This type of study is not applicable because it is a theoretical study.
