# Supplementary material for: Coefficient Estimates for New Subclasses of Bi-Univalent Functions Involving Generalized Bivariate Fibonacci-like Polynomials
Source: F1000Res. 2026 Apr 28;14:1463. Originally published 2025 Dec 26. [Version 2] doi: 10.12688/f1000research.173513.2 (PMC13216776; doi:10.12688/f1000research.173513.2)
Supplement: Peer review history

# Reviewer report: Reviewer response for version 2

**DOI:** 10.5256/f1000research.198846.r479969

**Published:** 2026-05-27

**Authors:** Matthew Olanrewaju Oluwayemi

**Affiliations:** 1 Nile University of Nigeria, Abuja, Nigeria

The authors have implemented the necessary corrections. Hence, the manuscript is recommended for indexing in its current form.

Is the work clearly and accurately presented and does it cite the current literature?

Yes

If applicable, is the statistical analysis and its interpretation appropriate?

Not applicable

Are all the source data underlying the results available to ensure full reproducibility?

No source data required

Is the study design appropriate and is the work technically sound?

Yes

Are the conclusions drawn adequately supported by the results?

Partly

Are sufficient details of methods and analysis provided to allow replication by others?

Yes

Reviewer Expertise:

NA

I confirm that I have read this submission and believe that I have an appropriate level of expertise to confirm that it is of an acceptable scientific standard.

# Reviewer report: Reviewer response for version 1

**DOI:** 10.5256/f1000research.191340.r459891

**Published:** 2026-03-09

**Authors:** Matthew Olanrewaju Oluwayemi

**Affiliations:** 1 Nile University of Nigeria, Abuja, Nigeria

**REVIEWER’S REPORT MANUSCRIPT ID:**

**Title:** Coefficient Estimates for New Subclasses of Bi-Univalent Functions Involving Generalized Bivariate Fibonacci-like Polynomials

**Author**: Husseinu M and H. Saloomi M  

**Journal:** F1000 Research

****

**In Review:**

**Title:** The title “Coefficient Estimates for New Subclasses of Bi-Univalent Functions Involving Generalized Bivariate Fibonacci-like Polynomials” agrees with the contents of the work.

**Abstract:** The abstract provides some fundamental information about the work. For instance, a brief background to the study in one sentence or two sentences and the knowledge gap. However, the purpose of the study, aim and objectives of the work, motivation, purpose and justification of the study, methodology and findings are missing. I therefore recommend that the authors should review or modify the abstract. 

 Introduction:  The authors considered coefficient estimates for certain classes of univalent functions (Bi-Univalent Functions) associated Generalized Bivariate Fibonacci-like Polynomials in the work using differential subordination. New and interesting results were presented in the study.

 Structural adjustment is also recommended in terms of the presentation. In particular, the following sections are necessary:

 1 **Introduction**: Introduction provided but requires some modifications and corrections as pointed out above. The authors need to provide sufficient background to the study and adequate review of relevant and current literature.

 2 **Preliminaries:** Section 2, titled “Method” should be the preliminaries. More so, Definition 3.1 and Remark 3.2 - Remark 3.5 should also come under the second section, titled “Preliminaries”.

 3 **Main Results:** The authors presented six (6) results in the work as follows, The section 3 titled “The family Γp,q,x,yΩ,Σ(X(ѵ)) and the coefficients bounds” and section 4, titled “Fekete-Szegö inequality for the class Mp,q,x,yΩ,Σ(X(ѵ))  and Γp,q,x,yΩ,Σ(X(ѵ))” should come under “Main Results”.  However, Definition 3.1 and Remark 3.2 - Remark 3.5 should come under “Preliminaries”.  The following results, Theorem 3.6, Theorem 4.3 and Theorem 4.6 with all associated corollaries should come under this section, section 3, titled “Main Results”.

**Theorem 3.6: **The word “Regarding” should be replaced with “if” or “For”

**Theorem 4.3: ** The phrase “Assuming that” should be replaced with “Let” or “Suppose”.  The proof of the Theorem should begin with an assumption.

**Theorem 4.6**:  The statement of the Theorem should be reviewed or modified. In particular, the phrase “Let suppose that” is tautology and rather be written as “Let” or “Suppose that” and not both.

 4 **Conclusion: **The conclusion in the work is not well presented by the authors. It should be well detailed. Hence, I recommend that it is modified.

**References: **The authors should follow the required reference style by arranging the references in the way and manner that they were cited in the body of the work. That is, [1], [2], [3], …and [14], [15] and [16]. The authors should also ensure that all cited papers are referenced and in the same manner, they also need to ensure that all reference papers are adequately cited in the body of the work. The references are current and relevant to some extent but need to be improved. Six (6) out of the fifteen (15) references representing 40% of the references are in the last ten (10) years of 2016 – 2026. I recommend that the authors update the references using the following: https://doi.org/10.3390/axioms12040360 (Ref 1); https://doi.org/10.1016/j.sciaf.2023.e01876 (Ref 2); https:// doi.org/10.3390/math12020169 (Ref 3); https://doi.org/10.3390/math14020292 (Ref 4); and any other relevant and current references.

**General comments**: The results are correct and scientific enough for indexing in the journal, F1000 journal. However, more review of background to the study and literature review is suggested. The references also need to be updated. The authors should consider the following references:

 1. https://doi.org/10.3390/ axioms12040360;

 2. https://doi.org/10.1016/j.sciaf.2023.e01876;

 3.      https:// doi.org/10.3390/math12020169;

 4. https://doi.org/10.3390/math14020292; and

 any other relevant and current papers

**Recommendation:** Recommended for indexing after necessary corrections.

Is the work clearly and accurately presented and does it cite the current literature?

Yes

If applicable, is the statistical analysis and its interpretation appropriate?

Not applicable

Are all the source data underlying the results available to ensure full reproducibility?

No source data required

Is the study design appropriate and is the work technically sound?

Yes

Are the conclusions drawn adequately supported by the results?

Partly

Are sufficient details of methods and analysis provided to allow replication by others?

Yes

Reviewer Expertise:

Complex analysis (Geometric Function Theory) and Numerical analysis (or Computational Mathematics)

I confirm that I have read this submission and believe that I have an appropriate level of expertise to confirm that it is of an acceptable scientific standard, however I have significant reservations, as outlined above.

## References

[1] : Coefficient Results concerning a New Class of Functions Associated with Gegenbauer Polynomials and Convolution in Terms of Subordination. *Axioms* .2023;12(4) : 10.3390/axioms12040360 10.3390/axioms12040360

[2] : Harmonic functions associated with Pascal distribution series. *Scientific African* .2023;21: 10.1016/j.sciaf.2023.e01876 10.1016/j.sciaf.2023.e01876

[3] : Bi-Univalency of m-Fold Symmetric Functions Associated with a Generalized Distribution. *Mathematics* .2024;12(2) : 10.3390/math12020169 10.3390/math12020169

[4] : Comprehensive Subfamilies of Bi-Univalent Functions Involving a Certain Operator Subordinate to Generalized Bivariate Fibonacci Polynomials. *Mathematics* .2026;14(2) : 10.3390/math14020292 10.3390/math14020292

# Response

**Published:** 2026-04-13

**Authors:** Mustafa Husseinu

**Affiliations:** Department of Mathematics, University of Kerbala, Karbala, Karbala Governorate, Iraq

Dear Reviewers,

 We would like to express our sincere gratitude for your careful review of our manuscript entitled:

 “Coefficient Estimates for New Subclasses of Bi-Univalent Functions Involving Generalized Bivariate Fibonacci-like Polynomials.”

 We truly appreciate your insightful comments and constructive suggestions, which have been invaluable in improving the quality, clarity, and overall presentation of our work.

 We have carefully revised the manuscript in accordance with all your comments. A summary of the major revisions is provided below:

 Abstract: The abstract has been completely revised to clearly present the background, aim, motivation, methodology, and main findings of the study.

 Introduction: The introduction has been expanded to include a stronger background and a more comprehensive review of recent and relevant literature.

 Manuscript Structure: The manuscript has been reorganized following your recommendations:

 The section previously titled “Method” has been renamed to “Preliminaries” and now includes all necessary definitions and remarks.

 The main results are now presented in a dedicated section titled “Main Results”.

 Theorems and their corresponding corollaries have been properly arranged.

 Language and Presentation: All suggested corrections related to wording and phrasing, especially in the statements of the theorems, have been carefully implemented.

 Conclusion: The conclusion has been significantly improved and expanded to better emphasize the importance of the results and highlight potential directions for future research.

 References: The references have been updated, reordered according to their appearance in the manuscript, and recent relevant studies have been added as recommended.

 We hope that the revisions adequately address all your comments and that the manuscript is now suitable for publication.

 Thank you once again for your valuable time and thoughtful feedback.

 Sincerely,

 Mustafa Husseinu

 Mohammed H. Saloomi

# Reviewer report: Reviewer response for version 1

**DOI:** 10.5256/f1000research.191340.r459885

**Published:** 2026-02-24

**Authors:** Alina Alb Lupas

**Affiliations:** 1 University of Oradea, Oradea, Romania

**REVIEWER’s REPORT**

*On the paper*

 Coefficient Estimates for New Subclasses of Bi-Univalent Functions Involving Generalized Bivariate Fibonacci-like Polynomials

 by Mustafa Husseinu, Mohammed H. Saloomi

 In this paper the authors introduce new subclasses of bi-univalent functions involving generalized Fibonacci polynomials and establish coefficient estimation and Fekete–Szegö inequalities for the functions belong to the defined subclasses.

 The results are new, correct and detailed. The paper is original and doesn’t contradict to ethical or policy issues, the question posed by authors is new and well defined, the methods used by authors are appropriate and well described, the data are sound and well controlled, the discussion and conclusions are well balanced, the title and abstract convey the obtained results, the writing is acceptable, the paper contains good scientific results.

 The paper doesn’t require a revision.

             Taking the above into consideration, I recommend the paper for Indexing.

Is the work clearly and accurately presented and does it cite the current literature?

Yes

If applicable, is the statistical analysis and its interpretation appropriate?

Not applicable

Are all the source data underlying the results available to ensure full reproducibility?

Yes

Is the study design appropriate and is the work technically sound?

Yes

Are the conclusions drawn adequately supported by the results?

Yes

Are sufficient details of methods and analysis provided to allow replication by others?

Yes

Reviewer Expertise:

Complex analysis

I confirm that I have read this submission and believe that I have an appropriate level of expertise to confirm that it is of an acceptable scientific standard.

# Response

**Published:** 2026-04-13

**Authors:** Mustafa Husseinu

**Affiliations:** Department of Mathematics, University of Kerbala, Karbala, Karbala Governorate, Iraq

Dear Reviewer,

 We sincerely thank you for your careful evaluation of our manuscript and for your positive and encouraging comments. We greatly appreciate your recognition of the originality, correctness, and scientific contribution of our work.

 Your valuable feedback is highly appreciated and encourages us to continue our research efforts.

 Sincerely,

 The Authors
